# “It’s on everyone’s plate”: a qualitative study into physicians’ perceptions of responsibility for smoking cessation

**DOI:** 10.1186/s13011-018-0186-x

**Published:** 2018-12-12

**Authors:** E. Meijer, M. Kampman, M. S. Geisler, N. H. Chavannes

**Affiliations:** Public Health and Primary Care, Leiden University Medical Center, Hippocratespad 21, PO Box 9600, 2300 RC Leiden, The Netherlands

**Keywords:** Physicians, Smoking cessation, Perceptions, Interviews, Framework approach

## Abstract

**Background:**

Little research has investigated in-depth how physicians perceive their role in smoking cessation care. This qualitative study sought to understand physicians’ perceptions of responsibility for smoking cessation.

**Methods:**

Data were collected through individual semi-structured interviews and focus group interviews between June and November 2017 in The Netherlands. We interviewed 5 addiction specialists, 5 anesthesiologist, 4 cardiologists, 8 GPs, 5 internists, 5 neurologists, 2 pediatricians, 6 pulmonologists, 7 surgeons, and 8 youth healthcare physicians (*N* = 55). Data analysis followed the framework approach.

**Results:**

The analysis showed that three actors were perceived as responsible for smoking cessation: physicians, patients, and the government. Participants perceived physicians as responsible for facilitating smoking cessation -albeit to different extents-, patients as carrying the ultimate responsibility for quitting smoking, and the government as responsible for creating a society in which smoking uptake is more difficult and quitting smoking easier. Perceptions of smoking itself were found to be important for how participants viewed responsibility for smoking cessation. It remained unclear for many participants which healthcare provider is responsible for smoking cessation care.

**Conclusions:**

The organization of smoking cessation care within health systems should be a focus of intervention, to better define physician roles and perceptions of responsibility. In addition, it seems important to target perceptions of smoking itself on the level of physicians and –as suggested by comments by several participants- the government.

**Electronic supplementary material:**

The online version of this article (10.1186/s13011-018-0186-x) contains supplementary material, which is available to authorized users.

## Background

Healthcare providers (HCPs) have an important role in facilitating smoking cessation. The World Health Organization for example stated that physicians should advise smokers to quit smoking [1], and physicians such as anesthesiologists and cardiologists have been encouraged to be more active in smoking cessation [2, 3]. Research shows that the majority of smokers wants to quit smoking, with health being the primary reason [4, 5]. Many smokers however are not advised to quit smoking when visiting their HCP [6–8]. In addition a range of effective smoking cessation interventions has been developed, including behavioral support, pharmacotherapy and eHealth [9–14], but these are underused [15]. As such, most quit attempts are unaided despite evidence that quit attempts that are supported by a HCP are more likely to succeed.

Many studies have investigated determinants of HCPs’ implementation of smoking cessation care (SCC), and show that factors at the level of the HCP (e.g. negative outcome expectancies, low self-efficacy), patient (e.g. lack of motivation to quit) and environment (e.g. lack of time, lack of referral possibilities) are important [6, 8, 16–32]. Although these factors are clearly important in themselves, it is also possible that physicians’ perceptions of their role in relation to smoking cessation underlie some of these factors. That is, physicians who do not perceive smoking cessation care as central to their role may be more likely to report that they do not have sufficient time for SCC, that other tasks interfere with SCC and so on. In line with this, role identity indeed emerged as a key HCP factor in a recent study among fourteen types of HCPs, such that HCPs who felt more responsible for providing SCC advised more smokers to quit smoking and had stronger intentions to use the Dutch SCC guideline, above and beyond the degree of barriers that they reported, such as lack of time, task interference, and lack of motivation to quit in patients [8].

Several studies show that many types of HCPs do not feel very responsible for smoking cessation [2, 33, 34]. Reasons for physicians’ perceptions of responsibility for smoking cessation have however not been studied much, although some studies have been conducted among physicians treating chronic obstructive pulmonary disease (COPD) patients. A multinational qualitative study shows that general practitioners (GPs) and pulmonologists did feel responsible for motivating patients with COPD to quit smoking, but were also frustrated by perceptions that COPD patients did not assume responsibility for quitting smoking [17]. Some physicians perceived smokers with COPD as ‘nonsensical’ and considered it to be their own fault that they had COPD, which made these physicians less inclined to prescribe expensive medication. As such, physicians’ sense of responsibility for SCC seems to relate to their perception of smokers and the extent to which smokers are responsible for quitting smoking. In line with this, many primary care physicians and pulmonologists in the United States perceived COPD as a ‘self-inflicted’ disease, and that one-third of them thought that COPD patients who continued to smoke could not be treated [35]. A study among German hospital physicians shows that over half of them believed that willpower alone was effective to quit smoking successfully, which is unlikely to facilitate provision of SCC [36].

Although it is important to understand how physicians perceive their role in SCC, as well as their patients’ responsibility for quitting smoking, little research has been conducted to investigate this. Studies among smokers themselves have shown that lung cancer and COPD patients avoid reporting symptoms due to perceptions of blame and stigma because of smoking [37, 38]. Similarly, smokers who perceive higher levels of smoking-related stigma are more likely to hide their smoking status from their HCP (see [39, 40] for a review of smoking-related stigma). Given that perceptions of blame and stigma affect smokers’ interactions with their HCPs, it is not unlikely that physicians’ perceptions of smoking and responsibility for smoking cessation affect their provision of SCC. The current qualitative study sought to understand physicians’ perceptions of responsibility for smoking cessation.

## Methods

### Design and participants

This study was part of a cross-sectional survey study into SCC by HCPs [8]. At the end of the survey, GPs and medical specialists were asked for permission to be approached for an interview about smoking cessation care and the Dutch Tobacco dependence guideline. Survey participants (*n* = 431) who provided permission were invited for an interview via e-mail between June 2017 and September 2017. Additional information on the interview was also provided (see Procedure). One hundred five physicians agreed to participate and 55 were selected to be interviewed, based on specialization (i.e., maximum of 8 participants per specialization) and -for practical reasons- reply date (i.e., those who responded first were scheduled to be interviewed). Interview participants were 5 addiction specialists, 5 anesthesiologist (1 in training), 4 cardiologists, 8 GPs (1 in training), 5 internists (2 in training), 5 neurologists, 2 pediatricians, 6 pulmonologists (1 in training), 7 surgeons, and 8 youth healthcare physicians (YHP). In the Netherlands addiction specialists and YHPs are physicians who have specialized in the treatment of addiction and patients under 18, respectively. In contrast to pediatricians, YHPs work in municipal organizations, schools, and consultation bureaus for infants.

### Procedure

Data were collected between June and November 2017 in The Netherlands. Before deciding on participation, potential participants received information on the study and were informed that participation is voluntary and can be ended at any time, that the interview would be audio-recorded, and that data would be used confidentially and anonymously for the purpose of scientific research only. They provided verbal informed consent (audio-recorded in a separate file) before the start of the interview. All interviews were semi-structured and conducted via telephone, except for one individual face-to-face interview with a participant who worked in the same center as the author, and two focus group interviews with 4 GPs and 4 YHPs, respectively (participant numbers 1–8, see Additional file 1 for the interview schedule). We initially aimed for face-to-face focus group interviews only, but this was not feasible given participants’ time constraints. The first author conducted the focus group interviews and 6 individual interviews, the second author conducted 37 individual interviews, and a trained Medicine master student conducted 4 individual interviews and assisted in focus group data collection. The second author and master student were trained by the first author to ensure quality of data collection. Individual interviews lasted 19 min on average excluding informed consent (range 9–42 min) and the focus group interviews with GPs and YHPs lasted 83 and 74 min, respectively. Data were collected until data saturation was reached. Interviews were recorded and transcribed verbatim. Participants received a € 20.- gift coupon for participation, and travel costs of focus group participants were reimbursed. The procedure was cleared for ethics by Leiden University Medical Center’s Medical Ethical Committee.

### Analysis

Data were analyzed according to the principles of the Framework approach [41, 42], which combines inductive and deductive analysis. As a first step, the first and second author familiarized themselves with four randomly selected transcripts, and independently coded the transcript using an initial coding tree that was developed based on literature [19, 28–32, 43, 44]. Both authors independently adapted the coding tree to capture relevant data, after which the initial analysis was discussed and the coding tree was finalized (see Additional file 2 for the final coding tree). The interviews were subsequently coded by the second and third author using Atlas.ti. The first author independently coded six transcripts, and the double-coded transcripts were discussed between the authors to ensure inter-coder reliability. The coding team was multidisciplinary (i.e. a psychologist and two Medicine master students, of whom two were nonsmokers and one currently smoked), which ensured that different viewpoints were captured. In general coding was found to be very similar between coders, and any discrepancies in coding were resolved through discussion. After coding, data for each participant were clustered in tables based on the codes. Finally, data from all participants were combined and interpreted to identify themes in the data in relation to the research question.

## Results

Fifty-five physicians were interviewed. On average participants were 45.73 years old (*SD* = 11.16), 29 participants were male (53%), and 45 had never smoked (82%), 16 were ex-smokers (16%) and 1 participant smoked. Smoking cessation was perceived as a shared responsibility between HCPs and patients, with patients having the ultimate responsibility for their own quit attempts. Many participants pointed out that the government should create an environment that facilitates successful quitting and lower smoking prevalence more generally.

### Physicians’ responsibility

Participants largely believed that physicians were responsible for facilitating smoking cessation. For example, a YHP mentioned that smoking ‘is clearly a serious health risk [...] in that sense I think it is definitely the responsibility of a doctor to talk about it’ (P17). Perceptions of smoking as disease also seemed to facilitate a sense of responsibility, and -as one cardiologist put it- ‘an addiction is a disease and that’s what we have doctors for’ (P54). However, although there was consensus that doctors should help smokers to quit smoking, different perspectives emerged on which physicians are responsible, and to what extent. This was not always clear, as an internist in training stated: ‘It’s not clear to me which task should be done by whom and how to arrange that’(P47). Several participants stated that every medical specialist should be engaged in SCC, as explained for example by this pulmonologist:

*“I feel that it is in fact a task for every healthcare provider, smoking is just bad for all kinds of things. It is not only bad for your lungs but also for your… it’s a high risk for cardiovascular disease, other malignancies, so it, it… it’s on everyone’s plate.”* (P32, pulmonologist).

In line with this quote, many participants recognized SCC as part of their responsibility. Participants appeared to perceive that the responsibility for SCC was not limited to physicians only (e.g. P32 quoted above ‘every HCP’).Most participants discussed responsibilities of their own and other specializations, and they considered their own specialization to be responsible for asking about smoking status and advising to quit smoking. However, around three-quarter of participants shifted the responsibility for counseling patients throughout their quit attempt to other healthcare professions or settings than their own, most often partially but sometimes entirely. For example, one neurologist stated that:

“*I think all of us* [physicians] *should raise awareness. I think it’s not really the task of say a neurologist to see people just for smoking cessation.* [...] *That type of care is very intensive. And it’s primarily aimed at preventing novel problems and, you know, as a specialist in a hospital you just don’t have the time*.” (P37, neurologist).

Whereas neurologists did not appear to perceive counseling as their task, several pulmonologists did but felt ill-equipped to counsel patients in their quit attempts:

*“As a pulmonologist you can’t offer it* [SCC] *because I’m just not experienced and I’m not allowed the time it takes. So you say yeah, it is super important that you quit smoking, and then well, figure it out for yourself.”* (P35, pulmonologist in training).

Like this pulmonologist, YHPs also considered their discipline responsible for referring to an expert because of ‘the complexity and relapses’ related to the process of smoking cessation (P5, YHP). Several addiction specialists agreed that ‘it is a specialist’s job’ (P52), and that ‘all the know-how is there’ (P34) within their specialization. Notably, although addiction specialists were generally perceived –by themselves and others- as the experts, they differed in the importance that they attached to smoking. That is, whereas one considered it ‘great if we would focus primarily on smoking cessation*’* (P34), his colleague stated that ‘in the work that I do, I cannot do that as well’ (P12). Many participants, including P12, suggested that smokers should be treated during a ‘specialized office hour at the general practice’ (P12). Overall, it appeared that general practice was perceived as the appropriate setting for SCC, both by medical specialists and the majority of GPs themselves. Practical reasons (e.g., accessibility, time, closer HCP-patient relationships) and general practice’s stronger focus on ‘preventive’ care played a role, although not all GPs agreed that they had enough time.

Several medical specialist regretted that more extensive SCC was not available in hospitals, which they thought was ‘strange’ (P43, pulmonologist in training), ‘a pity’ (P41, surgeon), or ‘very bad’ (P53, pediatrician). Some believed that SCC in secondary care would be more effective compared to primary care, such as the following pulmonologist:

*“When the GP refers the patient the patients thinks oh my, something’s really wrong! And that’s when you need to act. The patient that visits his GP twice a year and, you know, isn’t really bothered by his disease, he won’t readily admit that he’s ill.”* (P39).

There also appeared to be certain situations in which participants deliberately refrained from encouraging quitting based on the patients’ health condition. For example, a cardiologist did not advise patients to quit if ‘someone is 85 and has heart failure and kidney failure and lung problems […] I can’t make him live a hundred years anyway’ (P49)*.* Most participants in addition reported a limit to the physician’s responsibility based on the patients’ behavior, for example ‘if it [quitting smoking] has been discussed multiple times, at a certain point it’s done’ (P13, neurologist). Other participants, mostly GPs, reported encountering resistance or anger in patients, at which point they would refrain from providing SCC (P20, GP). This experience was described by some GPs as ‘tilting against windmills’ (P3) and ‘pushing a concrete wall’ (P13). Participants appeared to demarcate their sense of responsibility in order to protect themselves from ending up feeling disappointed, particularly when they did not expect their efforts to result in much progress, such as this surgeon explained:

“[Patients] *always just pretend like they learned incredibly much from my advice* [to quit]*.* […] *If he does not want to cooperate, I cannot make him, but then I can’t take responsibility either.”* (P20).

However, although many participants commented on a lack of motivation on the patients’ behalf, others stated that only a small minority (e.g. ‘5 to 10 percent’; P44, cardiologist) of patients is not motivated to quit.

### Patients’ responsibility

Participants considered both the patient and the HCP to be responsible for successful smoking cessation, with patients having the ultimate responsibility for their own quit success and for ‘taking good care of themselves’(P30, pulmonologist). The balance between the responsibilities of patients and HCPs was summarized as follows by an internist:

“*Taking medication, or quitting smoking, or following a program, yes that is of course the patient’s ultimate responsibility. But initiating* [a quit attempt] *and let’s say coaching and such like a GP can do, I find that really the task of a physician, or a different healthcare provider.”* (P28).

There were different perceptions of the extent of the patients’ responsibility for smoking cessation, which appeared to relate to perceptions of why people smoke. The quote of the following surgeon reflects a view on smoking that was shared by many participants:

*“I think that at a young age it is extremely encouraged and subsequently it is very addictive.”* (P41).

The addiction to smoking meant that, according to a neurologist, ‘people just cannot fix it themselves’ (P37, neurologist), suggesting that smokers cannot be held fully responsible for smoking if they started to smoke when young. A number of participants elaborated on the role of the tobacco industry as an important factor beyond the individual smoker’s control. One pulmonologist accounted how she explained this to her patients, particularly if they believed that they should be ‘strong enough’ to undertake an unaided quit attempt:

“*They* [cigarettes] *are made such that you don’t quit. You see, that’s of course the aim of the industry, to make sure that you keep buying them. (…) They* [patients] *will look at you like, oh, right, you have a point*.” (P9).

It did not always seem clear to HCPs how smoking should be perceived, for example one YHP stated that nurses wondered ‘is this someone’s own choice or not?’ (P5). Some participants had hybrid perceptions of smoking as both an addiction and a deliberate choice, such as the following YHP:

*“I think it’s a shame if people choose to continue smoking, but, well, addiction is a difficult thing.”* (P17, YHP).

Perceptions of smoking as the patient’s choice could either facilitate or hamper the provision of SCC. Some participants explained how they would use the patient’s own responsibility or choice to empower them in quitting smoking, as reflected in this GP’s account:

*“You want to give someone his personal responsibility back. […] Eventually there is one trigger, being that you apply it yourself.”* (P4).

However, for other participants it seemed that perceptions of continued smoking as a choice decreased their provision of SCC. In these cases choice, responsibility and fault appeared to be closely intertwined concepts. For example, one of the neurologists discussing an elderly patient in a nursing home explained:

*“I find it very preachy to say, it’s your own fault, it’s your own responsibility. So I won’t. I’ll say, it’s not healthy but you’ve been smoking for 70 years and those couple of years won’t make a difference.”* (P51).

Some participants who considered SCC as preachy brought up patients’ freedom or individual rights, such as this anesthesiologist:

*“For some people it* [smoking] *is something so essential. Freedom of expression. The right to smoke. And when a specialist patronizes like, my goodness, you should stop smoking, yes, that, that…* […] *not all people let themselves be lectured. And you do want to keep the relationship of trust intact.”* (P27).

Like this anesthesiologist, referring to the human right of freedom of expression, several other participants also related their views on smoking to political ideologies (e.g., ‘liberal’, P40, surgeon) or systems (‘a free country’, P30, pulmonologist).Whereas some participants who perceived smoking as a patient’s choice refrained from providing SCC, a few participants went a step further and more generally related continued smoking to provision of care. One of the neurologists expressed her frustration about patients who continue to smoke despite evident smoking-related complaints:

*“My goodness, so this patient returns in five years with a heart attack because he just continued smoking* […] *sometimes I feel reluctant to prescribe medication if people don’t do it* [quit smoking] […] *And of course patients differ so you will always have the procedure* [surgery] *but I do feel that, it wouldn’t have to be like this, if he had given up smoking.* […] *I think if I manage to help that patient quit smoking and it results in good healthy vessels, I did something good for that person.”* (P45, neurologist).

This sentiment was recognized by a number of surgeons and anesthesiologist (P21, P29, P38), although they considered it ‘ethically complicated’ to withhold treatment from a patient because of smoking. As such, it appeared that physicians at times struggled with the extent to which patients can be expected to take responsibility for smoking, and the consequences of this for their treatment regimen.

### Governmental responsibility

Several participants brought up the government’s responsibility for developing legislature that facilitates an optimal environment for SCC, and the role of society in preventing smoking uptake among youth. When asked whether SCC was a physician’s responsibility, a pediatrician responded as follows:

“*Partly. I think society also has a role in that. We* [physicians] *can’t prioritize it if there are so many factors in daily life that you know, do not discourage it* [smoking].” (P53).

The sentiment that societal changes are needed to optimize the effects of SCC was also reflected in the account of this GP:

*“So actually now we are doing the work, but if government would, say, prohibit smoking in many more places and increase taxes and make it more difficult to obtain cigarettes, then maybe we wouldn’t have to do a very large part of this work* [SCC].” (P15).

Participants considered campaigns aimed at preventing smoking uptake among adolescents, strong tax increases, and smoking bans in places such as schools, sports clubs, hospital to be helpful. Although there were some participants with positive perceptions of the role of the government when compared to the past (P22) and to other countries (P20), the predominant viewpoint was that the government was not sufficiently concerned with SCC. For example, a surgeon stated:

*“I am annoyed with how politics has been difficult about tobacco discouragement and that things are being reversed again. […] politics doesn’t look further than 4 years, because then there are new elections.”* (P16).

The following anesthesiologist believed that these financial considerations might prevent governmental intervention:

*“I find the government can be much more strict in this, but I still have the feeling that there is so much financial interest in it, that it restricts the government from putting all kinds of measures in effect.”* (P27).

In addition, the ideological considerations discussed above were also mentioned in relation to Dutch government. For example, a YHP commented that ‘Our minister of public health, who smokes, is very much pro individual freedom. So that’s not the best approach to steer this in the right direction.’ (P7)*.*

## Discussion

Many HCPs including physicians do not feel very responsible for SCC [2, 33, 36], but as yet the underlying reasons were largely unknown. Physicians views their own and the patients’ responsibilities for smoking cessation had also received little research attention. This qualitative study provided in-depth insight into physicians’ perceptions of responsibility for smoking cessation, including ten types of physicians. The analysis showed that physicians’ perceptions of smoking itself were important for how they viewed responsibility for smoking cessation. Furthermore, three actors were perceived as responsible for smoking cessation: physicians, patients, and the government. Participants perceived physicians as responsible for facilitating smoking cessation -albeit to different extents-, patients were perceived as carrying the ultimate responsibility for quitting smoking, and the government was perceived as responsible for creating a society in which smoking uptake is more difficult and quitting smoking more easy. These topics will be discussed in turn below.

With regard to physicians, several participants pointed out that every HCP should be involved in SCC. However if tasks are not clearly defined this may introduce shared responsibility bias [45, 46], resulting in fewer physicians in the end perceiving themselves as responsible for SCC. Results showed that most participants did consider it their own responsibility to advise smokers to quit. The extent of their sense of responsibility for counseling quit attempts differed however and many participants considered another group of physicians than their own, typically GPs or addiction specialists, to be better placed to counsel smokers. These views align with concise SCC protocols such as Ask-Advise-Refer and Ask-Advise-Connect [47], which underscore that whereas all HCPs should discuss smoking, they can refer for counseling rather than counsel smokers by themselves. Some GPs and addiction specialists did not feel that they should counsel smokers either, and other participants working in hospitals wanted SCC facilities within their own institution, such that there did not seem to be a coherent perspective on responsibilities of different types of physicians for SCC. Results also showed that participants perceived their responsibility for SCC to be limited by patients’ bad health status, in order not to cause distress to the patient, or by patient behavior that was perceived to indicate lack of motivation to quit smoking, in order not to become disappointed with negative outcomes themselves. This reflects findings of previous research which showed that HCPs often perceive smokers as lacking motivation to quit [8, 16], although studies among smokers have shown that most smokers actually want to quit smoking [4, 5]. Importantly, the perception that quitting smoking causes patient distress might be untrue and should be examined per patient group; for example a study among HCPs working with smokers with mental health problems shows that many HCPs erroneously believed that quitting smoking would worsen psychopathology [48].

With regard to responsibility of smokers, results showed that most participants believed that the majority of smokers had initiated smoking when young, after which they quickly became addicted. As such, most participants did not hold smokers fully responsible for their current smoking status. Some participants also mentioned the tobacco industry as an influence beyond the individual smoker’s control that stimulated them to continue smoking. However, smokers were perceived as having a key role in quitting smoking, and carrying the ultimate responsibility for quitting smoking. Participants thus believed that smokers had a choice in whether they would quit smoking or not. Conceptualizations of smoking as an addiction and a choice reflect recent debates in the literature on how smoking should be perceived [49]. Importantly, perceptions of choice could either hamper or facilitate participants’ provision of SSC. Whereas some participants underscored smokers’ choice in order to facilitate sense of agency in quitting, others appeared to equate choice and fault and were more reluctant to put effort into SCC when patients continued smoking. Some participants seemed to struggle with providing care in general to patients who continued to smoke, although none of the participants refrained from doing so. Such considerations have been reported before [17]. Participants sometimes related their perceptions of smoking as a choice to a ‘liberal’ ideology, or to the human right of ‘freedom of expression’. Ideological considerations were also apparent in participants’ discussion of the role of the government, which the large majority of participants who mentioned the government considered as insufficient. Some participants commented on the governments’ ‘liberal’ stance toward smoking, which they perceived as negative. Importantly, exactly these types of views on smoking have been identified as beneficial by the tobacco industry (i.e. Philip Morris) who wrote already in 1979 that ‘As the personal freedom concept is widely accepted and supported in Holland, the anti-smoking cause is not exceptionally strong. … Members of the medical profession and government appear to have highly individual opinions and the consensus is that smoking is a matter of personal choice.’ (cited in [50] p. 95). In line with this, academic scholars have also pointed out that such views on whether smoking is a voluntary behavior impact treatment and government policy [49].

The government emerged as a third actor. Several participants stated that physicians’ efforts are not enough if the government does not develop legislature that facilitates an optimal environment for SCC. Participants appeared to believe that conflicts of interest with regard to money or own smoking status prohibited some government officials from taking action on smoking. In line with evidence on antismoking measures, participants perceived campaigns, tax increases and smoking bans as effective in reducing smoking prevalence [51–53], and were generally positive about instigation of these measures.

This study has limitations. First, it is possible that physicians who were interested in the topic of SCC were more inclined to respond quickly to the invitation and to participate in the interview. Participants of the current qualitative study were recruited through a large-scale online survey, for which we approached physicians and other HCPs regardless of their experience with SCC, thereby aiming to mitigate risk of bias. However, as is inherent to qualitative research, results are not meant to be generalized to all physicians in The Netherlands or beyond. Quantitative studies are needed to examine whether the perceptions that emerged in the current study are representative, whether these vary between different types of physicians and other HCPs, and which characteristics (HCP, organization, patient, etc.) are related to these perceptions. For example, a study among addiction treatment providers in the United Stated and United Kingdom shows that differences in beliefs about addiction are related to HCP characteristics such as age, own addiction history, workplace and spiritual beliefs [54]. It would be interesting to examine this for views on responsibility for smoking cessation as well. Second, participants may have provided socially desirable answers, although they were ensured that data would be analyzed and reported anonymously. Given that several participants expressed views that may be somewhat controversial, we do not expect social desirability to have played an important role. Third, we used two methods to collect data in order to include a sufficient number of participants, which may have led to slightly different participant responses. Whereas individual telephonic interviews are relatively anonymous and allow for one participant to express his view, face-to-face focus groups are less anonymous and allow for interaction between participants. In addition, conclusions drawn from the focus group interviews with GPs and YHPs were very similar to conclusions based on individual interviews with participants from these respective groups.

## Conclusions

It remained unclear for many participants which physician or HCP is responsible for SSC, suggesting that the organization of SCC needs to be improved. Results suggest that an infrastructure in which every HCP advises smokers to quit and then refers them to specialized smoking cessation HCPs (such as specialized nurses or addiction specialists with more complex cases) is feasible, and may facilitate physicians’ sense of responsibility. It also seems important to target perceptions of smoking itself on the level of physicians and –as suggested by comments by several participants- the government, such that they perceive smoking as an addiction with severe health consequences, rather than a somewhat innocent habit that smokers choose to engage in [55]. This may stimulate the development of legislation that facilitates smoking cessation, and strengthen physicians’ perceptions of their responsibility.

## Additional files

Additional file 1: Interview protocol. (DOCX 18 kb)

Additional file 2: Final coding tree. (DOCX 18 kb)

## Abbreviations

COPD: Chronic obstructive pulmonary disease
GP: General practitioner
HCP: Healthcare provider
P35: Participant (e.g. 35)
SCC: Smoking cessation care
YHP: Youth healthcare physician
