# Supplementary material for: “It’s on everyone’s plate”: a qualitative study into physicians’ perceptions of responsibility for smoking cessation
Source: Subst Abuse Treat Prev Policy. 2018 Dec 12;13:48. doi: 10.1186/s13011-018-0186-x (PMC6290505; doi:10.1186/s13011-018-0186-x)
Supplement: Supplementary file 1 — Interview protocol. (DOCX 18 kb) [file 13011_2018_186_MOESM1_ESM.docx]

**Additional file 1: Interview protocol**

*Activate the voice-recorder and put the phone on speaker mode.*

1. Introduction, overview of the research, ask for informed consent
   1. Thank you for participating in this interview. In addition to the questionnaire, we would like to go deeper into your experiences with smoking cessation care and the guideline. As a thank you for your participation you will receive a 20 ERUOS voucher.
   2. Before we start, I would like to discuss some matters with you. This telephone interview will be recorded. All the information you give us will be used confidentially and anonymously for scientific research purposes only. You will not be identifiable in any publications. We have previously sent you this information by email. Have you received and understood this information, and do you agree with participation in this study on these terms?

*Start a new audio file such that informed consent and interview are two separate files.*

1. Demographic data
   1. I would like to start with a number of general questions about your work. How many patients do you see per week?

i. How many of them do you see in an outpatient clinic environment?

- 1. What percentage of these patient smokes tobacco?

1. Can you tell me something about your experiences with smoking cessation care?
   1. Do you ask your patients if they smoke? When, when not?
   2. Do you undertake action if patients smoke (quit advice, assess motivation to quit etc.)?

i. If so, what kind of action do you take?

ii. How much time do you spend on this in the consultation?

- 1. What helps you to provide smoking cessation care?

i. Factors concerning yourself?

ii. Factors concerning the patient?

iii. Factors concerning your working environment?

- 1. Are there things that make it difficult to provide smoking cessation care? Can you tell me something about that?

i. Factors concerning yourself?

ii. Factors concerning the patient?

iii. Factors concerning your working environment?

iv. Own smoking behavior:

- Do you smoke, or have you ever smoked?

If participant is a current smoker:

- Does this affect whether or not you assess the patient's smoking status? And whether you provide smoking cessation care? Please explain.

If participant is an ex-smoker:

- For how long have you quit smoking?

- Has something changed since then in what you do regarding smoking cessation? Please explain.

- 1. Do you see smoking cessation care as the responsibility of a physician? Why yes / no? If not, who is responsible?

i. What place does smoking cessation have in your specialization?

ii. Do you consider smoking cessation care an important part of your work?

iii. Do you consider this task part of your own specialism or do you find it a task for another specialist / care provider? Why?

- 1. How do you find the organization of smoking cessation care in the Netherlands?
  2. What do you think of smokers?

i. Why do people smoke, according to you?

ii. Why do your patients smoke, according to you?

1. Are you familiar with the ‘Tobacco Dependence’ guideline?

IF 4 = YES

a. What are your experiences with the ‘Tobacco Dependence’ guideline?

b. Do you know the content of the guideline?

i. Is the content clear to you?

ii. Are there things you miss in the guideline?

iii. Do you apply the guideline? If so, how?

iv. Does the guideline help you to provide better care?

c. What do you think are positive aspects of the guideline?

d. Do you have points for improvement for the guideline?

IF 4 = NO

e. Is there another guideline or protocol that you use? Which guideline / protocol is this?

IF 4E = General practitioners’ ‘Stop Smoking’ guideline

i. Is the content clear to you?

ii. Are there things that you miss in the guideline?

iii. Do you apply the guideline? If so, how?

iv. Does the guideline help you to provide better care?

f. What do you think are positive aspects of the guideline?

g. Do you have points for improvement for the guideline?

IF 4E = Yes, different

h. What are your experiences with this guideline / protocol? What do you think are positive aspects of the guideline? Do you also have points for improvement?

1. Final remarks and completing the survey:

- Thank you for participating in this interview. Are there any other things you would like to say about providing smoking cessation care or about the guideline?

*- Ask for address where gift coupon can be sent and write this down*
