# Supplementary material for: “It’s on everyone’s plate”: a qualitative study into physicians’ perceptions of responsibility for smoking cessation
Source: Subst Abuse Treat Prev Policy. 2018 Dec 12;13:48. doi: 10.1186/s13011-018-0186-x (PMC6290505; doi:10.1186/s13011-018-0186-x)
Supplement: Supplementary file 2 — Final coding tree. (DOCX 18 kb) [file 13011_2018_186_MOESM2_ESM.docx]

**Additional file 2: Final coding tree**

1. Demographic data
   1. Own smoking status/history
      1. Influence on smoking cessation care
2. Smoking cessation counselling (SCC) tasks
   1. Ask about smoking status
   2. Advise to quit
   3. Assess motivation to quit
   4. Increase motivation to quit
   5. Assist in quitting
   6. Arrange follow-up
   7. Prescribe/advise medication or NRT
   8. Advise about e-cigarette
   9. Refer patients
   10. Return to smoking in later consultation
   11. Provide information/education
   12. Other SCC tasks
3. Overall evaluation of providing smoking cessation counselling
   1. Positive
   2. Negative
   3. Best way of providing SSC
4. Barriers to providing SCC
   1. Own
      1. Motivation
      2. Knowledge
      3. Skills
      4. Self-efficacy
      5. Outcome expectations
      6. Lack of training
      7. Smoking status
      8. Observability
      9. Not appropriate to talk about it
      10. Other ‘own’ barriers
   2. Patients
      1. Unmotivated
      2. Unhelpful social environment
      3. Comorbidity/multiple problems
      4. Expectations
      5. Demographic patient characteristics
      6. Resistance to SSC
      7. Dishonest about smoking behaviour
      8. Lack of knowledge about the effects of smoking
      9. Other ‘patients’ barriers
   3. Environment
      1. Lack of facilities
      2. Lack of time
      3. Other tasks
      4. Lack of social support
      5. Lack of materials
      6. Lack of reimbursement
      7. Lack of referral possibilities
      8. Other ‘environment’ barriers
5. Facilitators of providing SCC
   1. Own
      1. Motivation
      2. Knowledge
      3. Skills
      4. Self-efficacy
      5. Outcome expectations
      6. Demarcated sense of responsibility
      7. Training
      8. Smoking status
      9. Observability
      10. Other ‘own’ facilitators
   2. Patients
      1. Motivation
      2. Smoking-related complaints
      3. Demographic patient characteristics
      4. Other ‘patients’ facilitators
   3. Environment
      1. Facilities
      2. Time
      3. Social support
      4. Materials
      5. Reimbursement
      6. Referral possibilities
      7. Other ‘environment’ facilitators
6. Responsibility for smoking cessation care
   1. Medical doctor
      1. Limits of responsibility
   2. Patient
   3. Both medical doctor and patient
   4. Government
   5. Society
   6. Other
7. Place of SCC in healthcare
   1. Fit SSC with own profession
   2. Fit SSC with other profession
   3. Motivates colleagues for SSC
   4. Perception of colleagues’ SSC activities
8. Smoking cessation care/measures in the Netherlands
   1. Organization of care
      1. Fragmented/negative
      2. Structured/positive
      3. Unfamiliar with it/unclear
   2. Evaluation of antismoking measures
      1. Increase taxes
      2. Education at schools
      3. Campaigns
      4. Smoking ban in public places
      5. Fewer tobacco selling points
      6. Reward system for non-smokers
      7. Complete ban on tobacco
      8. Increase age for buying tobacco
      9. Other measures
9. Government tasks
   1. Government is sufficiently involved
   2. Government is not sufficiently involved
   3. Prevention can be better
10. Perception of smoking
    1. Smoking is a choice
    2. Smoking is own responsibility
    3. Smoking is an addiction
    4. Smoking is a disease
    5. Opinion about smokers
    6. Smokers’ reasons for smoking
       1. Upbringing
       2. Social environment/peer group pressure
       3. Habit
       4. Relaxation
       5. Boredom
       6. Addiction
       7. Culture-dependent
       8. Other reasons
11. Guideline
    1. Guideline usage
    2. Content
       1. Positive
       2. Negative
    3. Relative advantage
    4. Compatibility
    5. Complexity
    6. Trialability
    7. Observability
    8. Other guideline aspects
    9. Uses GP-guideline ‘Stop smoking’
       1. Content
          1. Positive
          2. Negative
       2. Relative advantage
       3. Compatibility
       4. Complexity
       5. Trialability
       6. Observability
       7. Other guideline aspects
    10. Uses other protocol
12. Tobacco industry
